# Quantification of edge-enhancing effects using accelerated deep learning reconstructed orbital MRI sequences^☆^

**DOI:** 10.1016/j.ejro.2026.100761

**Published:** 2026-05-15

**Authors:** Christer Ruff, Deborah Staber, Georg Gohla, Carina Kelbsch, Frank Paulsen, Daniel Vogl, Constantin Roder, Ulrike Ernemann, Till-Karsten Hauser

**Affiliations:** a Department of Diagnostic and Interventional Neuroradiology, Eberhard Karls University Tuebingen, Tuebingen D-72076, Germany; b Centre for Ophthalmology, University Eye Hospital, Eberhard Karls University of Tuebingen, Tuebingen D-72076, Germany; c Department of Radiation Oncology, University Hospital Tuebingen, Tuebingen D-72076, Germany; d Department of Neurosurgery, University of Tuebingen, Tuebingen D-72076, Germany; e Center for Neuro-Oncology, Comprehensive Cancer Center Tuebingen-Stuttgart, University Hospital of Tuebingen, Eberhard Karls University of Tuebingen, Tuebingen D-72070, Germany

**Keywords:** Orbit, Magnetic resonance imaging, Deep learning reconstruction, Turbo spin-echo, Edge sharpness, Blurriness

## Abstract

**Rationale and Objectives:**

To qualitatively and quantitatively compare image quality, edge sharpness, and internal-structure delineation of accelerated deep learning-reconstructed turbo spin-echo (TSE_DLR_) sequences versus conventionally reconstructed TSE (TSE_CR_) in 3 T orbital MRI.

**Materials and Methods:**

Twenty-five patients undergoing 3 T orbital MRI were retrospectively selected. All underwent conventional and accelerated (up to 75%) deep learning-reconstructed axial and coronal T2-weighted and contrast-enhanced fat-saturated T1-weighted TSE (T1CEfs) sequences. Two blinded neuroradiologists rated image quality, edge sharpness, internal tissue delineation, overall sharpness, lesion detection confidence, and lesion conspicuity on a 5-point Likert scale. Quantitative analyses included similarity and image-quality metrics, the Perceptual Sharpness Index (PSI), blur metrics, and edge parameters after Roberts, Sobel, or Canny edge extraction.

**Results:**

Qualitatively, edge and overall sharpness were superior with TSE_DLR_ in T1CEfs (all p < 0.001), and mainly in T2-weighted imaging. Internal tissue delineation was rater-dependent, with the experienced rater favoring TSE_CR_. Lesion-detection confidence did not differ significantly across reconstructions. Quantitatively, TSE_DLR_ improved edge sharpness, with increased edge steepness (all p < 0.001) and decreased edge width (most p ≤ 0.003), though edge contrast was slightly reduced. Blur metrics favored TSE_CR_, while PSI (all p < 0.001) and SNR/PSNR favored TSE_DLR_. Structural similarity was high (SSIM 0.986–0.995; MS-SSIM 0.998–0.999), while high-frequency similarity was lower (FSIM: 0.804–0.861; WASH: 0.729–0.747).

**Conclusion:**

Accelerated DL-based reconstructions for orbital MRI maintain or improve lesion conspicuity, overall image quality, and edge sharpness, while substantially reducing scan time. However, reduced internal anatomical delineation warrants cautious interpretation of the images.

## 1 Introduction

High-quality orbital MRI is essential for evaluating intrabulbar and intraorbital pathology, including optic nerve involvement, and requires thin-slice, high-resolution imaging with a sufficient signal-to-noise ratio (SNR) [1]. Traditional acceleration methods, such as parallel imaging and compressed sensing, can shorten scan times but risk degrading image quality when pushed to higher acceleration factors [2]. Radial acquisition sequences offer motion robustness but may compromise fat suppression and spatial resolution, leading to blurred margins, which can be attributed to lower sampling density in the peripheral regions of k-space [1], [2], [3], [4]. Deep learning reconstruction (DLR) methods address these trade-offs by using data-driven upsampling to enhance effective matrix size and image resolution by inferring high-frequency information. These techniques have been implemented clinically across multiple anatomical regions, including the liver, prostate, brain, and spine [1], [2], [3], [5]. However, orbital MRI studies remain rare [4].

Prior DLR studies predominantly focus on simple quantitative image quality measures, such as improved SNR and contrast-to-noise ratio (CNR), greater perceived image quality, and sharper images relative to conventional reconstructions (CR) [6], [7]. But DLR can also produce more homogeneous tissue representations that experienced radiologists perceive as blurred, and therefore, might result in inferior anatomical delineation, as demonstrated in a recent neuroradiological study of the hippocampus, brainstem, and cerebellum [8]. Although standardized methods exist to quantify, for example, SNR and CNR, increased image sharpness is typically reported only qualitatively in radiological studies of accelerated DLR MRI [9], [10], [11], [12]. Edge sharpness, i.e., the rate and extent of intensity transitions at anatomical boundaries, is an essential but underreported metric. Numerous sharpness methods have been proposed, but not all translate well to MRI. Non-parametric gradient- or kurtosis-based approaches are noise-sensitive and unreliable at low SNR [13], [14], [15], [16]. Similarly, rise distance (10–90%) or the full width at half maximum (FWHM) distance are affected by noise and do not fully utilize the full available data [17], [18], [19]. Parametric edge modeling techniques are more noise-robust and have been applied to quantify sharpening in optical imaging and to assess margin sharpness in CT [20], [21].

The purpose of this study was to compare both qualitatively and quantitatively accelerated deep learning-reconstructed turbo spin-echo (TSE_DLR_) sequences with conventionally reconstructed turbo spin-echo (TSE_CR_) sequences for T2-weighted (T2) and contrast-enhanced, fat-saturated T1-weighted (T1CEfs) orbital MRI, with specific focus on edge sharpness quantified via edge steepness, edge width, and edge contrast, and blurriness.

## 2 Materials and methods

### 2.1 Study design

This retrospective, IRB-approved, single-center study (IRB No. 549/2024BO2) was conducted in accordance with the Declaration of Helsinki. Twenty-five patients underwent clinical 3 T orbital MRI between April and November 2023.

### 2.2 Imaging protocol and deep learning reconstruction algorithm

All examinations were performed on a 3 T clinical scanner (MAGNETOM Vida Fit, Siemens Healthineers, Erlangen, Germany) using a 20-channel head coil. Table 1 shows sequence parameters for coronal and axial T2-weighted and contrast-enhanced fat-saturated T1-weighted TSE (T1CEfs) sequences for conventionally (TSE_CR_) and as undersampled DLR variants (TSE_DLR_).Conventional TSE used an acceleration factor of 2 phase-encoding (PE) steps, whereas DLR used 4 PE steps, reducing acquisition time by up to 75%. Reconstruction was performed offline on standard MRI workstation hardware. The DLR algorithm, Deep Resolve Boost (Siemens Healthineers, Erlangen, Germany), was cleared by the U.S. Food and Drug Administration (FDA) and has been applied in neuroradiological and non-neuroradiological contexts [22], [23], [24], [25]. It employs a variational network approach that alternates between physics-based data-consistency steps and learned convolutional neural network (CNN) regularization within a fixed, unrolled cascade. The CNN component, referred to as the "Deep, Iterative, Hierarchical Network", extends the Down-Up architecture by introducing nested hierarchical blocks, enabling multi-resolution processing while conserving memory. A separate calibration acquisition provides coil sensitivity maps and a bias field estimate to ensure homogeneous image intensity.

**Table 1:** Imaging parameters.

Parameter	T2_CR_ ax	T2_DLR_ ax	T2_CR_ cor	T2_DLR_ cor	T1CEfs_CR_ ax	T1CEfs_DLR_ ax	T1CEfs_CR_ cor	T1CEfs_DLR_ cor
Field of view, mm^2^	160	160	160	160	160	160	160	160
Voxel size, mm^3^	0.2 × 0.2 × 2.0	0.2 × 0.2 × 2.0	0.2 × 0.2 × 3.0	0.2 × 0.2 × 3.0	0.3 × 0.3 × 2.0	0.3 × 0.3 × 2.0	0.3 × 0.3 × 3.0	0.3 × 0.3 × 3.0
Slice thickness, mm	2	2	3	3	2	2	3	3
Number of slices	23	23	34	34	23	23	34	34
Base Resolution	352	352	352	352	256	256	272	272
Parallel imaging factor	2	4	2	4	2	4	2	4
Acceleration mode	GRAPPA	GRAPPA	GRAPPA	GRAPPA	GRAPPA	GRAPPA	GRAPPA	GRAPPA
Repetition time, ms	5350	5350	3790	3790	647	647	628	628
Echo time, ms	86	86	86	86	10	10	10	10
Averages	1	1	1	1	2	2	2	2
Concatenations	1	1	2	2	2	2	3	3
Acquisition time, min	2:47	0:42	3:11	0:50	2:43	1:07	5:06	1:38
Time savings using TSE_DLR_ sequences, min (%)		2:05 (75%)		2:21 (74%)		1:36 (59%)		3:28 (68%)
Total time savings using TSE_DLR_ sequences, min (%)	4:17 (69%)							

CR, conventional reconstruction; DLR, deep learning reconstructed technique; ax, axial; cor, coronal; CE, contrast-enhanced; fs, fat-saturated; GRAPPA, GeneRalized Autocalibrating Partial Parallel Acquisition (parallel imaging technique); TSE, Turbo Spin-Echo; mm, millimeter; ms, millisecond; min, minute

### 2.3 Qualitative image quality assessment

Two blinded neuroradiologists with 1 year (Rater 1) and 10 years (Rater 2) of experience independently evaluated anonymized datasets in random, mixed order across separate sessions, with the reconstruction type concealed, and at least a 2-week washout period between sessions to mitigate bias [26]. Readings were performed in certified reading room conditions on a dedicated workstation (GE Centricity PACS RA 1000, version 7.0.2; General Electric (GE) Healthcare, Chicago, Illinois, USA) and certified diagnostic radiology monitors (RadiForce RX350, Eizo Corporation, Hakusan, Ishikawa, Japan).

The raters evaluated the datasets using a 5-point Likert scale, with 5 representing the highest quality. Raters scored overall image quality, edge sharpness, internal tissue delineation ("blurriness"), and overall sharpness. When lesions were present, lesion conspicuity and detection confidence were rated separately. The detailed scoring definitions for each category are provided in Supplementary Table 1.

### 2.4 Quantitative similarity and image quality metrics

Image quality metrics (IQMs) were computed using MATLAB R2024b (The MathWorks, Inc., Natick, MA, USA). Structural fidelity of DLR images relative to CR reference images was assessed with Structural Similarity Index (SSIM) and Multi-Scale SSIM (MS-SSIM) [27], [28]. The Feature Similarity Index (FSIM) captured the fidelity of high-frequency components [29]. Wavelet-Based Sharp Features (WASH) is an image quality assessment metric based on the human visual system that accounts for human vision's sensitivity to image sharpness and zero-crossings [30]. Signal-to-noise ratio (SNR) and peak SNR (PSNR) were also calculated using MATLAB's built-in functions [28], [31]. The Perceptual Sharpness Index (PSI) is a no-reference metric estimating perceived image sharpness from the statistical properties of local edge gradients, incorporating aspects of human visual perception [32]. PSI continuously decreases as edge sharpness declines.

### 2.5 Quantification of blur

Blur was assessed by using one no-reference metric and one reference metric [28], [33], [34]. The reference metric for blurriness provides a percentage value (the blur percentage; higher values indicate greater blur) [33]. The non-reference perceptual blur metric ranges from 0 to 1, defining the best and the worst quality in terms of blur perception, respectively [34]. The latter quantifies blur by low-pass filtering the image and measuring changes in neighboring pixel intensities. Larger changes indicate a sharper original image, while smaller changes suggest pre-existing blur.

### 2.6 Quantification of edge sharpness

**Fig. 1:**
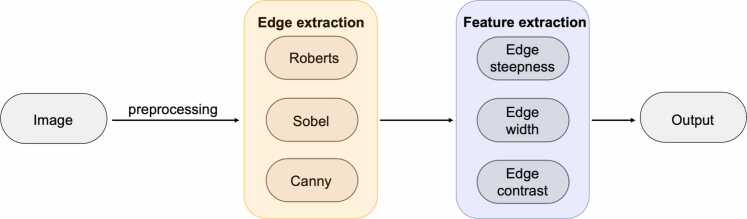
Illustration of the multilayer network workflow for edge sharpness calculations. After using one of the edge extraction methods (Roberts, Sobel, or Canny), feature extraction is performed, including edge sharpness, edge width, and edge contrast.

**Fig. 2:**
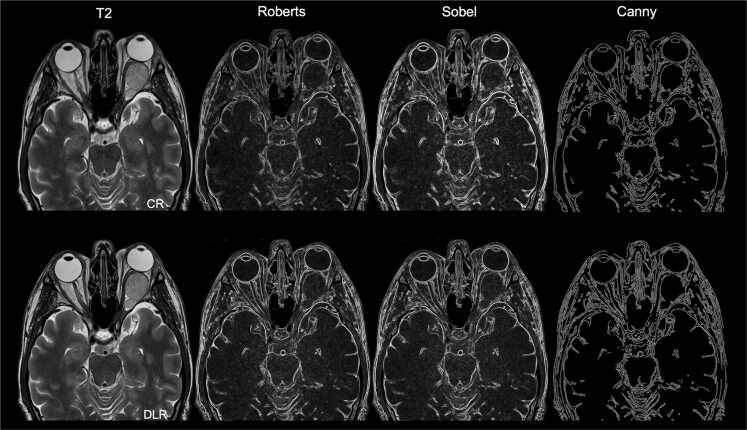
Exemplary representation of the Roberts, Sobel, and Canny edge extraction methods for an axial conventional and deep learning reconstructed T2-weighted TSE image. CR, conventional reconstruction; DLR, deep learning reconstructed technique.

**Fig. 3:**
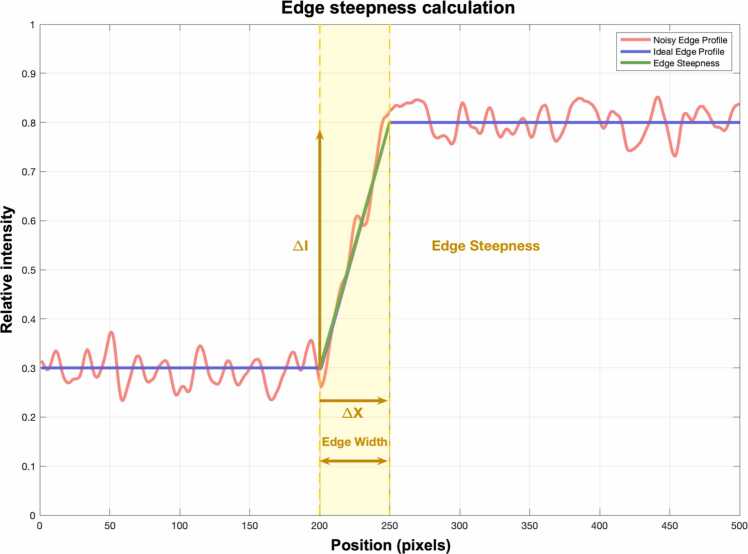
Schematic illustration of edge steepness quantification. Edge steepness quantifies the rate of intensity change across an edge. It is calculated as the maximum magnitude of the local gradient in a small neighborhood around each edge pixel. First, the horizontal and vertical gradients are computed using the MATLAB gradient() function. As edges are rarely perfectly horizontal or vertical, gradient magnitude is calculated using the Euclidean length of the gradient vector (gradientMagnitude = max $\sqrt{G_{x}^{2}+\;G_{y}^{2}}$) where Gx and Gy are the horizontal and vertical components of the gradient obtained using central differences, resulting in a rotationally invariant measure of image gradients. Higher steepness values indicate sharper edges with a more abrupt transition. I, intensity.

**Fig. 4:**
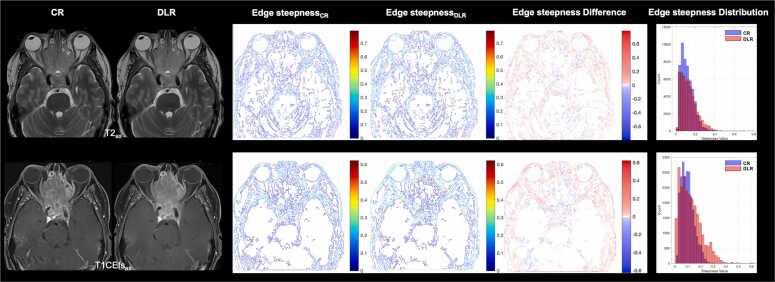
Exemplary axial images of a 47-year-old male patient with a histologically confirmed embryonal rhabdomyosarcoma (T4b) for edge steepness calculations. Feature extractions, including edge steepness, are performed for conventional (CR) and deep learning (DL) reconstructed axial (ax) T2-weighted (T2) and contrast-enhanced fat-saturated T1-weighted (T1CEfs) TSE images to quantitatively assess edge sharpness using an edge extraction method such as Canny. DL-reconstructed images exhibit greater edge steepness than conventional reconstructions, with higher steepness values indicating sharper edges and more abrupt transitions. CR, conventional reconstruction; DLR, deep learning reconstructed technique; CE, contrast-enhanced; fs, fat-saturated; ax, axial.

**Fig. 5:**
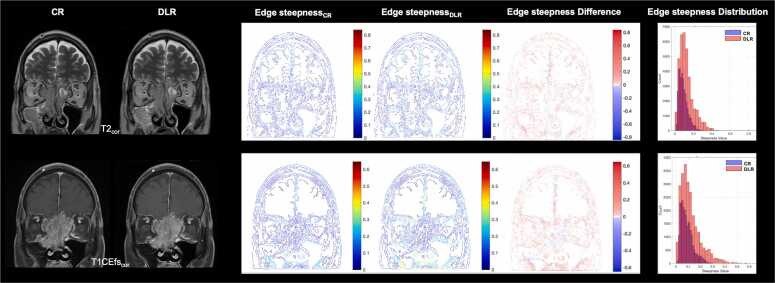
Exemplary coronal images of a 47-year-old male patient with a histologically confirmed embryonal rhabdomyosarcoma (T4b) for edge steepness calculations. Feature extractions, including edge steepness, are performed for conventional (CR) and deep learning (DL) reconstructed coronal (cor) T2-weighted (T2) and contrast-enhanced fat-saturated T1-weighted (T1CEfs) TSE images to quantitatively assess edge sharpness after using an edge extraction method such as Canny. DL-reconstructed images exhibit greater edge steepness than conventional reconstructions, with higher steepness values indicating sharper edges and more abrupt transitions. CR, conventional reconstruction; DLR, deep learning reconstructed technique; CE, contrast-enhanced; fs, fat-saturated; cor, coronal.

Quantification of edge sharpness was performed by calculating mean edge steepness, mean edge width, and mean edge contrast using an intensity profile line with a homemade MATLAB R2024b (The MathWorks, Inc., Natick, MA, USA) script. First, the image is preprocessed using one of the edge-extraction models: Roberts, Sobel, or Canny. The aforementioned metrics are then used for feature extraction (Fig. 1**).** Fig. 2 shows exemplary images of the Roberts, Sobel, and Canny edge extraction methods.Edge steepness quantifies the rate of change in intensity across an edge. It is calculated as the maximum magnitude of the local gradient in a small neighborhood around each edge pixel. First, the horizontal and vertical gradients are computed using the MATLAB gradient() function. Since edges are rarely perfectly horizontal or vertical, the gradient magnitude is calculated using the Euclidean length of the gradient vector (gradientMagnitude = max $\sqrt{G_{x}^{2}+\;G_{y}^{2}}$) where G_x_ and G_y_ are the horizontal and vertical components of the gradient obtained using central differences. This results in a rotationally invariant measure of image gradients. Higher steepness values indicate sharper edges with more abrupt transitions (Fig. 3). Quantification examples of edge steepness for T2-weighted and T1CEfs images are shown in Fig. 4 for TSE_ax_ and in Fig. 5 for TSE_cor_.

Edge width is defined as the number of pixels over which the image intensity rises from 10% to 90% of its full contrast across an edge. Therefore, a sharp edge has a smaller edge width (fast transition), whereas a blurred edge tends to have a larger edge width (slow transition). Mean edge width is simply the average of all measured edge widths across detected edges in the image.

Edge contrast is the difference between the maximum and minimum intensity in a local neighborhood around an edge. High contrast edges tend to have a significant intensity change (e.g., white vs. black), whereas low contrast edges tend to have a subtle intensity change (e.g., light gray vs. dark gray). Mean edge contrast is the average edge contrast across all detected edges.

### 2.7 Statistical analysis

Statistical analyses were performed in SPSS Statistics Version 30 (IBM, Armonk, New York, USA). Continuous variables were presented as mean ± standard deviation (SD), and ordinal variables as median and interquartile range (IQR). Qualitative image comparisons between groups were analyzed using the Wilcoxon signed-rank test. Interrater reliability and concordance were assessed using Cohen's kappa and Kendall's tau-b and tau-c. The Cohen's coefficient d effect sizes were interpreted as follows: negligible to slight |d| = 0.01–0.2; fair |d| = 0.21–0.40; moderate |d| = 0.41–0.60; substantially large |d| = 0.41–0.60; and almost perfect agreement |d| = 0.81–1.00 [35]. The interpretation of Kendall's effect sizes and concordance was conducted under the following criteria: non = 0 < 0.10; fair = 0.10 < 0.30; moderate = 0.30 < 0.50; large = 0.50 < 0.70; substantially large to almost perfect agreement = 0.70 < 1.00. Paired *t*-tests were performed for non-reference objective image quality metrics and edge sharpness assessments between CR and DRL. Measurement variability was summarized using the coefficient of variation (CV): CV = [the within-participant standard deviation]/within-participant mean × 100%. Agreement across edge detectors was evaluated using a two-way mixed-effects model of intra-class correlation (ICC) for consistency and absolute agreement [36]. Higher ICC values indicate stronger reliability, as described by Shrout and Fleiss, Cicchetti, and Koo et al. [36], [37], [38]. The significance level was set at alpha = 0.05.

## 3 Results

The characteristics of the 25 included patients are presented in Table 2. The cohort had a mean age of 59.8 ± 19.9 years and comprised 18 males and 7 females. The most common referral indication was tumor-related (11/25, 44%), followed by inflammation-related (10/25, 40%). Visible pathology was present in 17/25 cases (68%).

**Table 2:** Patient demographics and characteristics.

Characteristics	Values
Number of examinations	n = 25
Age, yrs	59.8 ± 19.9
Sex	
Male Female	18 (72%) 7 (28%)
Reasons for assignment	
Tumor-associated Choroidal melanoma Squamous cell carcinoma Rhabdomyosarcoma Lacrimal gland tumor Morbus Hodgin Cavernoma Paget’s disease Exclusion of tumor/optic nerve compression	n = 11 n = 3 n = 1 n = 1 n = 1 n = 1 n = 2 n = 1 n = 1
Inflammation-associated Optic neuritis Tolosa-Hunt syndrome Endocrine orbitopathy Unclear etiology. presumably in the context of a COVID-19 infection	n = 10 n = 6 n = 2 n = 2 n = 1
Ischemia-associated Anterior ischemic optic neuropathy (AION)	n = 3 n = 3
Other causes Bilateral optic atrophy unclear etiology	n = 1 n = 1

Data are means ± standard deviations, the number of patients with the percentage in parentheses.

Yrs, years

### 3.1 Qualitative assessment of patient images

Qualitative ratings are summarized in Table 3 (T2-weighted sequences) and Table 4 (T1CEfs-weighted sequences). Representative images are provided in Fig. 4, Fig. 5, respectively. For T2-weighted imaging, TSE_DLR_ was associated with higher sharpness, particularly in the coronal plane. Edge sharpness improved significantly in most comparisons, except for Rater 1 in T2_ax_ (4 [4,5] vs. 5 [4,5], p = 0.206). Overall sharpness ratings consistently favored DLR (all p ≤ 0.012). Internal tissue delineation was inconsistent with Rater 1 preferring DLR for T2_cor_ (p = 0.004), whereas Rater 2 assigned lower DLR scores for T2_ax_ (4 [4,5] vs. 4 [3.5–4], p = 0.013). Overall image quality improvement reached significance only for Rater 2. Detection confidence did not differ between reconstructions (all p ≥ 0.317). For T1CEfs imaging, DLR produced significant improvements in edge and overall sharpness for both raters and orientations (all p < 0.001). Image quality was generally superior with DLR, except for axial acquisitions rated by Rater 1 (5 [4,5] vs. 5 [4.5–5], p = 0.132). Internal tissue delineation again diverged between raters with Rater 1 favoring DLR (both p < 0.001), while Rater 2 assigned lower DLR scores (axial 4 [4] vs. 4 [3,4], p = 0.007; coronal 4 [3,4] vs. 4 [3,4], p = 0.029). Detection confidence did not differ (all p ≥ 0.180).

**Table 3:** Comparison of qualitative ratings of the overall image quality, and the image sharpness of conventional and deep learning reconstructed T2-weighted images by two neuroradiologists with one year (Rater 1) and ten years (Rater 2) of experience. Pooled results are given for each category. The p-values were calculated using the Wilcoxon signed-rank test.

Image features		T2_CR_ ax		T2_DLR_ ax			T2_CR_ cor		T2_DLR_ cor		
		Mdn (IQR)	M ± SD	Mdn (IQR)	M ± SD	p-value	Mdn (IQR)	M ± SD	Mdn (IQR)	M ± SD	p-value
Overall Image Quality	Rater 1	5 [4−5]	4.76 ± 0.66	5 [4.5–5]	4.72 ± 0.54	0.655	5 [4−5]	4.52 ± 0.65	5 [4−5]	4.64 ± 0.57	0.317
	Rater 2	5 [4−5]	4.48 ± 0.71	5 [5−5]	4.84 ± 0.37	0.029	4 [4−5]	4.24 ± 0.60	5 [4.5–5]	4.76 ± 0.44	< 0.001
Edge sharpness	Rater 1	4 [4−5]	4.24 ± 0.83	5 [4−5]	4.40 ± 0.82	0.206	4 [3−4]	3.44 ± 0.71	4 [4−5]	4.44 ± 0.51	< 0.001
	Rater 2	4 [4−5]	4.12 ± 0.60	5 [4−5]	4.60 ± 0.58	0.001	4 [3−4]	3.52 ± 0.59	4 [4−5]	4.52 ± 0.51	< 0.001
Delineation of internal tissue structures (“blurriness”)	Rater 1	4 [4−5]	4.00 ± 1.08	5 [4−5]	4.28 ± 1.06	0.115	3 [3−4]	3.40 ± 0.65	5 [3.5–5]	4.12 ± 1.13	0.004
	Rater 2	4 [4−5]	4.12 ± 0.73	4 [3.5–4]	3.72 ± 0.79	0.013	4 [4−4]	3.92 ± 0.49	4 [3−4]	3.64 ± 0.64	0.07
Overall sharpness	Rater 1	4 [4−5]	4.04 ± 0.98	5 [4−5]	4.44 ± 0.71	0.012	4 [2–4.5]	3.48 ± 0.59	4 [4−5]	4.24 ± 0.52	< 0.001
	Rater 2	4 [4−5]	4.08 ± 0.70	5 [4−5]	4.64 ± 0.57	< 0.001	4 [3–4.5]	3.56 ± 0.51	4 [4−5]	4.40 ± 0.50	< 0.001
Confidence of detection	Rater 1	4 [2.5–5]	3.71 ± 1.31	4 [2.5–5]	3.71 ± 1.16	1	4 [2–4.5]	3.29 ± 1.45	4 [1.5–4.5]	3.24 ± 1.52	0.655
	Rater 2	4 [3−5]	4.06 ± 0.83	4 [3.5–5]	4.12 ± 0.78	0.317	4 [2.5–4.5]	3.88 ± 0.78	4 [3−5]	3.94 ± 0.83	0.564
Conspicuity of the lesion	Rater 1	4 [2–4.5]	3.35 ± 1.41	4 [2–4.5]	3.47 ± 1.28	0.317	3 [2–4.5]	3.00 ± 1.41	4 [2−4]	3.18 ± 1.38	0.317
	Rater 2	4 [3–4.5]	3.71 ± 1.05	4 [3.5–4.5]	4.00 ± 0.71	0.096	3 [2.5–4.5]	3.29 ± 1.21	4 [3.5–4]	3.76 ± 0.83	0.021

ax, axial; cor, coronal; CR, conventional reconstruction; DLR, deep learning reconstruction; Mdn, median; IQR, interquartile range; M, mean; SD, standard deviation.

**Table 4:** Comparison of qualitative ratings of the overall image quality, and the image sharpness of conventional and deep learning reconstructed T1-weighted (T1CE) images by two neuroradiologists with one year (Rater 1) and ten years (Rater 2) of experience. Pooled results are given for each category. The p-values were calculated using the Wilcoxon signed-rank test.

Image features		T1CEfs_CR_ ax		T1CEfs_DLR_ ax			T1CEfs_CR_ cor		T1CEfs_DLR_ cor		
		Mdn (IQR)	M ± SD	Mdn (IQR)	M ± SD	p-value	Mdn (IQR)	M ± SD	Mdn (IQR)	M ± SD	p-value
Overall Image Quality	Rater 1	5 [4−5]	4.52 ± 0.65	5 [4.5–5]	4.72 ± 0.54	0,132	5 [4−5]	4.40 ± 0.76	5 [5−5]	4.76 ± 0.52	0,003
	Rater 2	4 [4−5]	4.40 ± 0.58	5 [5−5]	4.80 ± 0.41	0,004	4 [4−5]	4.36 ± 0.70	5 [5−5]	4.8 ± 0.41	< 0,001
Edge sharpness	Rater 1	3 [3−4]	3.32 ± 0.48	5 [4−5]	4.56 ± 0.58	< 0.001	3 [3−4]	3.36 ± 0.64	4 [4−5]	4.36 ± 0.64	< 0,001
	Rater 2	3 [3−4]	3.44 ± 0.51	5 [4−5]	4.64 ± 0.49	< 0.001	3 [3−4]	3.44 ± 0.51	4 [4−5]	4.48 ± 0.59	< 0,001
Delineation of internal tissue structures (“blurriness”)	Rater 1	4 [3−4]	3.44 ± 0.71	4 [4−5]	4.40 ± 0.65	< 0,001	3 [3−4]	3.32 ± 0,63	4 [4−5]	4.12 ± 0.88	< 0,001
	Rater 2	4 [4−4]	3.96 ± 0.61	4 [3−4]	3.60 ± 0.50	0.007	4 [3−4]	3.92 ± 0.70	4 [3−4]	3.56 ± 0.58	0.029
Overall sharpness	Rater 1	3 [3−4]	3.48 ± 0.51	4 [4−5]	4.36 ± 0.57	< 0.001	3 [3−4]	3.40 ± 0,58	4 [4−5]	4.28 ± 0.68	< 0,001
	Rater 2	3 [3−4]	3.56 ± 0.51	4 [4−5]	4.52 ± 0.59	< 0.001	4 [3−4]	3.52 ± 0,51	5 [4−5]	4.40 ± 0.71	< 0,001
Lesions (n = 17) Confidence of detection	Rater 1	4 [3−5]	3.65 ± 1.32	4 [3−5]	3.82 ± 1.29	0,180	4 [2.5–5]	3.53 ± 1,37	4 [2−5]	3.47 ± 1.62	0,257
	Rater 2	4 [3−5]	3.94 ± 0.83	4 [3−5]	4.12 ± 0.86	0,180	4 [3−5]	3.88 ± 0,99	5 [3−5]	4.06 ± 1.14	0,180
Conspicuity of the lesion	Rater 1	4 [2−5]	3.47 ± 1.33	4 [2.5–5]	3.44 ± 1.42	0,414	4 [2–4.5]	3.41 ± 1,28	5 [2−5]	3.71 ± 1.65	0,739
	Rater 2	4 [3−5]	3.76 ± 0.97	4 [3−5]	4.00 ± 0.88	0,046	4 [3−5]	3.76 ± 0.97	4 [3−5]	4.00 ± 1.12	0,102

ax, axial; cor, coronal; CR, conventional reconstruction; DLR, deep learning reconstruction; CE, contrast-enhanced; Mdn, median; IQR, interquartile range; M, mean; SD, standard deviation.

### 3.2 Interrater intraprotocol agreement of image quality-based analysis

Detailed interrater statistics are provided in Supplementary Table 2. In summary, consensus was greater for DLR than for CR on overall image quality and edge sharpness. Cohen's κ improved with DLR across all sequences for image quality (e.g., for T2_ax_ it increased from 0.388 to 0.636 and for T1CEfs_cor_ it increased from 0.800 to 0.878). Edge sharpness agreement was similarly strong (e.g., for T1CEfs_ax_ it increased from 0.647 to 0.836). Agreement on blurriness was low and inconsistent across both methods (κ value range from −0.080–0.649). Agreement on overall sharpness was mixed, with κ decreasing in some DLR sequences.

### 3.3 Quantitative similarity and image quality assessment

IQM results are reported in Table 5**.** SSIM and MS-SSIM values were close to 1 (on a 0–1 scale) across all sequences (e.g. SSIM range 0.986–0.995), indicating near-perfect structural similarity. Conversely, FSIM (0.804–0.861) and WASH (0.729–0.747) were lower, suggesting differences in high frequency feature representation. SNR and PSNR were consistently higher for DLR images, aligning with the improved qualitative ratings. Using the PSI as a non-reference metric, substantial disparities were identified between CR and DLR across all sequences and orientations, with lower values for CR (p < 0.001 for all), indicating reduced sharpness.

**Table 5:** Results of similarity and image quality metric (IQM) analysis with conventionally reconstructed (CR) and deep learning reconstructed (DLR) MRI sequences for reference and non-reference metrics. For reference metrics, CR serves as reference.

	Sequence	IQM	value (M ± SD)	p-value
Reference metrics CR vs. DLR	T2_ax_	SSIM	0.9952 ± 0.0025	n.a.
		MS-SSIM	0.9993 ± 0.0004	n.a.
		FSIM	0.8610 ± 0.0304	n.a.
		WASH	0.7472 ± 0.0132	n.a.
		SNR	14.5259 ± 1.7372	n.a.
		PSNR	58.7483 ± 1.6232	n.a.
		Blur percentage	0.1167 ± 0.0776	n.a.
	T2_cor_	SSIM	0.9862 ± 0.0128	n.a.
		MS-SSIM	0.9979 ± 0.0019	n.a.
		FSIM	0.8035 ± 0.0693	n.a.
		WASH	0.7383 ± 0.0214	n.a.
		SNR	11.7167 ± 2.9024	n.a.
		PSNR	55.8629 ± 3.0594	n.a.
		Blur percentage	0.5820 ± 0.3677	n.a.
	T1CEfs_ax_	SSIM	0.9945 ± 0.0020	n.a.
		MS-SSIM	0.9993 ± 0.0002	n.a.
		FSIM	0.8164 ± 0.0245	n.a.
		WASH	0.7387 ± 0.0113	n.a.
		SNR	12.3692 ± 1.0747	n.a.
		PSNR	59.3844 ± 1.0931	n.a.
		Blur percentage	0.2297 ± 0.0840	n.a.
	T1CEfs_cor_	SSIM	0.9943 ± 0.0021	n.a.
		MS-SSIM	0.9994 ± 0.0002	n.a.
		FSIM	0.8210 ± 0.0305	n.a.
		WASH	0.7290 ± 0.0135	n.a.
		SNR	10.3893 ± 1.3322	n.a.
		PSNR	59.8547 ± 1.4334	n.a.
		Blur percentage	0.4205 ± 0.0919	n.a.
Non-reference metrics	T2_ax_	PSI_CR_	0.3585 ± 0.0182	< 0.001
		PSI_DLR_	0.4214 ± 0.0130	
		Blur metric_CR_	0.4231 ± 0.0280	< 0.001
		Blur metric_DLR_	0.3680 ± 0.0197	
	T2_cor_	PSI_CR_	0.2984 ± 0.0240	< 0.001
		PSI_DLR_	0.3939 ± 0.0151	
		Blur metric_CR_	0.5057 ± 0.0709	< 0.001
		Blur metric_DLR_	0.3475 ± 0.0542	
	T1CEfs_ax_	PSI_CR_	0.2645 ± 0.0041	< 0.001
		PSI_DLR_	0.3389 ± 0.0073	
		Blur metric_CR_	0.4506 ± 0.0197	< 0.001
		Blur metric_DLR_	0.3384 ± 0.0189	
	T1CEfs_cor_	PSI_CR_	0.2180 ± 0.0135	< 0.001
		PSI_DLR_	0.2991 ± 0.0154	
		Blur metric_CR_	0.5297 ± 0.0590	< 0.001
		Blur metric_DLR_	0.4088 ± 0.0554	

Data are presented as mean ± standard deviation. IQM, Image Quality Metric; M, mean; SD, standard deviation; ax, axial; cor, coronal; CR, conventional reconstruction; DLR, deep learning reconstruction; CE, contrast-enhanced; fs, fat-saturated; SSIM, Structural Similarity Index; MS-SSIM, Multi-Scale SSIM; FSIM, Feature Similarity Index; NQM, Noise Quality Metric; SNR, signal-to-noise ratio; PSNR, peak signal-to-noise ratio; WASH, Wavelet Based Sharp Features; PSI, Perceptual Sharpness Index.

### 3.4 Quantitative blur assessment

**Fig. 6:**
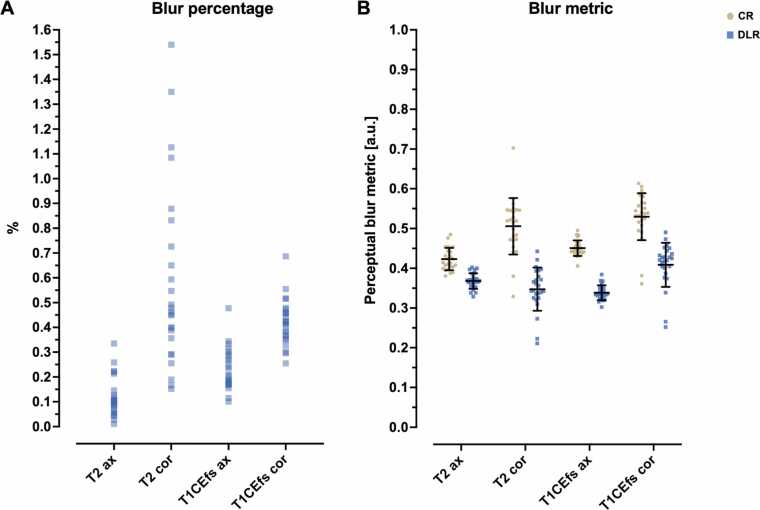
Comparison of image quality metrics, blur percentage (A), and perceptual blur metric (B) for conventional (CR) and deep learning reconstructed (DLR) axial (ax) and coronal (cor) T2-weighted and contrast-enhanced fat-saturated T1-weighted (T1CEfs) TSE images. Using blur percentage as a reference metric, with CR as the reference, indicates slightly greater blurring in DLR. This is evident in the percentages: 0% represents an identical image, and 100% represents a completely blurred comparison image. The no-reference perceptual blur metric, ranging from 0 to 1, represents the best and the worst quality in terms of blur perception. The metric shows lower DLR values, indicating greater blurriness. ax, axial; cor, coronal; a.u., arbitrary unit; CR, conventional reconstruction; DLR, deep learning reconstructed technique; CE, contrast-enhanced; fs, fat-saturated.

Results of quantitative blur assessments are shown in Table 5**.** Blur percentage showed only slight differences between DLR and CR serving as the reference image, indicating more blur for DLR images. It yielded the following results: 0.1167 ± 0.0776 for T2_ax_, 0.5820 ± 0.3677 for T2_cor_, 0.2297 ± 0.0840 for T1CEfs_ax_, and 0.4205 ± 0.0919 for T1CEfs_cor_ (Fig. 6**A**). The no-reference blur metric exhibited statistically significant disparities between CR and DLR across all sequences and imaging planes (p < 0.001 for all). Specifically, CR yielded higher blur-metric values than DLR, indicating greater image blurring for DLR (Fig. 6**B**).

### 3.5 Quantification of edge sharpness

**Fig. 7:**
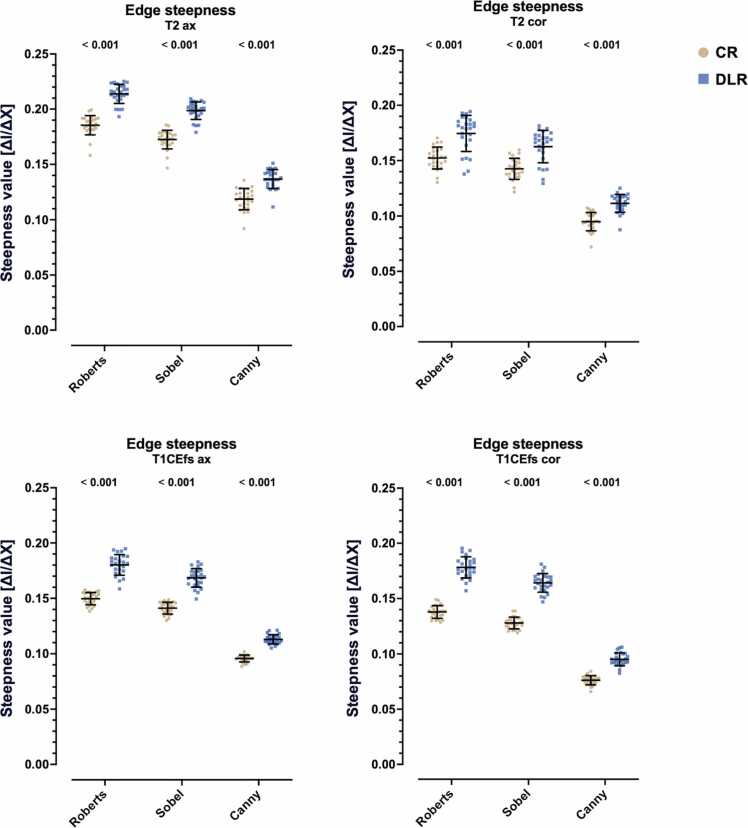
Comparison of edge steepness calculations for conventional (CR) and deep learning reconstructed (DLR) axial (ax) and coronal (cor) T2-weighted and contrast-enhanced fat-saturated T1-weighted (T1CEfs) TSE images for the edge extraction methods Roberts, Sobel, and Canny. Regardless of the selected edge detection method, the edge steepness values for DLR are higher, indicating sharper edges with more abrupt transitions. ax, axial; cor, coronal; a.u., artificial unit; CR, conventional reconstruction; DLR, deep learning reconstructed technique; CE, contrast-enhanced; fs, fat-saturated; I, intensity, X, horizontal position (pixels).

A detailed analysis of edge sharpness parameters is provided in Table 6 and Fig. 7. Across all evaluated sequences, DLR yielded a significantly higher mean edge steepness than CR for each edge detector (Roberts, Sobel, and Canny; all p < 0.001). For example, in T2-weighted axial imaging, mean edge steepness increased from 0.185 ± 0.056 (CR) to 0.214 ± 0.071 (DLR) using Roberts detection with comparable increases for Sobel and Canny. Similarly, Roberts-based edge steepness calculations for T2_cor_ increased from 0.152 ± 0.042 (CR) to 0.175 ± 0.070 (DLR), and for T1CEfs_cor_ from 0.138 ± 0.049 (CR) to 0.178 ± 0.081 (DLR) (all p < 0.001), suggesting a consistent increase in intensity transitions at anatomical boundaries with DLR.

**Table 6:** Results of edge sharpness parameter analysis with conventionally reconstructed (CR) and deep learning reconstructed (DLR) MRI sequences.

	Sequence		Edge sharpness parameter	value (M ± SD)	p-value
Mean edge steepness	T2_ax_	Roberts	Edge steepness_CR_	0.1854 ± 0.0563	< 0.001
			Edge steepness_DLR_	0.2139 ± 0.0714	
		Sobel	Edge steepness_CR_	0.1725 ± 0.0576	< 0.001
			Edge steepness_DLR_	0.1987 ± 0.0722	
		Canny	Edge steepness_CR_	0.1187 ± 0.0618	< 0.001
			Edge steepness_DLR_	0.1367 ± 0.0760	
	T2_cor_	Roberts	Edge steepness_CR_	0.1523 ± 0.0424	< 0.001
			Edge steepness_DLR_	0.1746 ± 0.0700	
		Sobel	Edge steepness_CR_	0.1426 ± 0.0448	< 0.001
			Edge steepness_DLR_	0.1626 ± 0.0711	
		Canny	Edge steepness_CR_	0.0950 ± 0.0516	< 0.001
			Edge steepness_DLR_	0.1114 ± 0.0682	
	T1CEfs_ax_	Roberts	Edge steepness_CR_	0.1497 ± 0.0692	< 0.001
			Edge steepness_DLR_	0.1802 ± 0.0682	
		Sobel	Edge steepness_CR_	0.1411 ± 0.0450	< 0.001
			Edge steepness_DLR_	0.1685 ± 0.0692	
		Canny	Edge steepness_CR_	0.0957 ± 0.0514	< 0.001
			Edge steepness_DLR_	0.1129 ± 0.0710	
	T1CEfs_cor_	Roberts	Edge steepness_CR_	0.1379 ± 0.0493	< 0.001
			Edge steepness_DLR_	0.1782 ± 0.0809	
		Sobel	Edge steepness_CR_	0.1279 ± 0.0511	< 0.001
			Edge steepness_DLR_	0.1685 ± 0.0692	
		Canny	Edge steepness_CR_	0.0762 ± 0.0510	< 0.001
			Edge steepness_DLR_	0.0951 ± 0.0749	
Mean edge width	T2_ax_	Roberts	Edge width_CR_	3.8138 ± 0.0708	0.157
			Edge width_DLR_	3.7639 ± 0.2013	
		Sobel	Edge width_CR_	3.6449 ± 0.0577	0.144
			Edge width_DLR_	3.6041 ± 0.1542	
		Canny	Edge width_CR_	3.4974 ± 0.0780	< 0.001
			Edge width_DLR_	3.4111 ± 0.1068	
	T2_cor_	Roberts	Edge width_CR_	3.7236 ± 0.0651	< 0.001
			Edge width_DLR_	3.4846 ± 0.3266	
		Sobel	Edge width_CR_	3.6041 ± 0.1542	0.003
			Edge width_DLR_	3.3571 ± 0.3019	
		Canny	Edge width_CR_	3.4366 ± 0.0685	< 0.001
			Edge width_DLR_	3.2958 ± 0.1496	
	T1CEfs_ax_	Roberts	Edge width_CR_	3.6730 ± 0.0416	< 0.001
			Edge width_DLR_	3.6730 ± 0.0416	
		Sobel	Edge width_CR_	3.5175 ± 0.0346	< 0.001
			Edge width_DLR_	3.3659 ± 0.1002	
		Canny	Edge width_CR_	3.3992 ± 0.0385	< 0.001
			Edge width_DLR_	3.1730 ± 0.0596	
	T1CEfs_cor_	Roberts	Edge width_CR_	3.6342 ± 0.0489	< 0.001
			Edge width_DLR_	3.3802 ± 0.1138	
		Sobel	Edge width_CR_	3.4666 ± 0.0402	< 0.001
			Edge width_DLR_	3.2468 ± 0.1311	
		Canny	Edge width_CR_	3.3409 ± 0.0438	< 0.001
			Edge width_DLR_	3.1620 ± 0.0662	
Mean edge contrast	T2_ax_	Roberts	Edge contrast_CR_	0.5494 ± 0.0192	< 0.001
			Edge contrast_DLR_	0.5362 ± 0.0186	
		Sobel	Edge contrast_CR_	0.5186 ± 0.0192	< 0.001
			Edge contrast_DLR_	0.5096 ± 0.0150	
		Canny	Edge contrast_CR_	0.3549 ± 0.0254	0.216
			Edge contrast_DLR_	0.3523 ± 0.0230	
	T2_cor_	Roberts	Edge contrast_CR_	0.5235 ± 0.0246	< 0.001
			Edge contrast_DLR_	0.4697 ± 0.0422	
		Sobel	Edge contrast_CR_	0.4956 ± 0.0237	< 0.001
			Edge contrast_DLR_	0.4471 ± 0.0385	
		Canny	Edge contrast_CR_	0.3269 ± 0.0240	< 0.001
			Edge contrast_DLR_	0.3113 ± 0.0204	
	T1CEfs_ax_	Roberts	Edge contrast_CR_	0.4745 ± 0.0138	< 0.001
			Edge contrast_DLR_	0.4330 ± 0.0210	
		Sobel	Edge contrast_CR_	0.4480 ± 0.0132	< 0.001
			Edge contrast_DLR_	0.4137 ± 0.0192	
		Canny	Edge contrast_CR_	0.2983 ± 0.0088	< 0.001
			Edge contrast_DLR_	0.2748 ± 0.0129	
	T1CEfs_cor_	Roberts	Edge contrast_CR_	0.4648 ± 0.0126	< 0.001
			Edge contrast_DLR_	0.4522 ± 0.0205	
		Sobel	Edge contrast_CR_	0.4339 ± 0.0122	0.003
			Edge contrast_DLR_	0.4259 ± 0.0175	
		Canny	Edge contrast_CR_	0.2543 ± 0.0109	0.001
			Edge contrast_DLR_	0.2429 ± 0.0175	

Data are presented as mean ± standard deviation. M, mean; SD, standard deviation; ax, axial; cor, coronal; CR, conventional reconstruction; DLR, deep learning reconstruction; CE, contrast-enhanced; fs, fat-saturated.

Regarding mean edge width, DLR generally exhibited lower values (i.e., narrower transitions consistent with increased sharpness), with significant reductions in most sequence/method combinations. Notably, the edge width of T2_ax_ did not differ significantly between CR and DLR with either the Roberts (p = 0.157) or Sobel (p = 0.144) methods. However, the Canny method detected a modest yet significant reduction (3.497 ± 0.078 (CR) vs. 3.411 ± 0.107 (DLR), p < 0.001). T2_cor_ exhibited consistent and significant decreases in edge width with DLR across all detectors (all p ≤ 0.003). In T1CEfs sequences, DLR was associated with smaller edge widths across all detectors (all p ≤ 0.001).

For mean edge contrast, DLR yielded contrast values that were marginally lower than those from CR. These differences were significant in the majority of comparisons (p < 0.001 in most). For instance, in T2_cor_, Roberts-based edge contrast decreased from 0.524 ± 0.025 (CR) to 0.470 ± 0.042 (DLR, p < 0.001), and in T1CEfs_ax_, it decreased from 0.475 ± 0.014 (CR) to 0.433 ± 0.021 (DLR, p < 0.001). An exception was observed for edge contrast of T2_ax_ using Canny, with no significant difference (p = 0.216).

### 3.6 Variability and agreement across edge detection methods

The variability and agreement across edge sharpness metrics (edge steepness, edge width, and edge contrast), and edge detection methods (Roberts, Sobel, and Canny) are presented in Supplementary Table 3. Across sequences, within-participant coefficients of variation (CV) indicated generally low-to-moderate dispersion across edge detection methods, with CVs for mean edge steepness ranging from 3.3% to 9.4%. Higher variability was particularly evident in coronal DLR datasets. Similar findings were observed for edge width and edge contrast.

Method-to-method consistency was high for edge steepness, with single-measure ICCs ranging from 0.778 to 0.947 and average-measure ICCs ranging from 0.913 to 0.982, supporting good-to-excellent rank-order agreement across detectors. However, ICC for steepness was substantially lower regarding absolute agreement, indicating systematic offsets between detectors despite strong consistency. Regarding edge width, ICCs for consistency were more modest than for steepness, indicating moderate-to-good consistency across detectors. Absolute agreement was generally limited (with average ICCs often in the low-to-moderate range). However, one notable exception was DL-accelerated coronal T2-weighted imaging, where absolute agreement for edge width was comparatively high (average ICC 0.901). For mean edge contrast, ICCs for consistency indicated moderate-to-excellent agreement across methods, whereas ICCs for absolute agreement remained low, consistent with detector-dependent scaling differences in contrast estimates.

## 4 Discussion

DLR is increasingly integrated into routine clinical MRI and is a key approach for overcoming the limitations of traditional acceleration methods while improving diagnostic performance and patient comfort [39], [40]. However, real-world experience and direct comparisons with CR sequences are still evolving. Consequently, we performed a comprehensive qualitative and quantitative comparison of DLR and CR in orbital MRI, with particular focus on the frequently overlooked metric of edge sharpness. Prior work suggests that radiologists' preferences for DLR depend on level of experience and knowledge, daily routine, and familiarity, and are influenced by differences in image sharpness and impression, among other things [41]. Ruff et al. have shown that, in a multidisciplinary neuro-oncology setting, qualitative ratings of DLR sequences can be improved to varying degrees. However, these ratings may differ depending on the discipline and the daily use of imaging or sequences [25]. In our study, both raters consistently preferred DLR images for edge sharpness across all sequences, and DLR maintained or improved overall image quality. These qualitative ratings were supported by higher SNR and PSNR for DLR, consistent with prior studies [42], [43], [44], [45], [46]. As DLR images typically exhibit lower noise levels than those obtained using traditional full-scan techniques, this trait can make DLR images appear unnatural to experienced radiologists [47], [48]. The DLR algorithm tends to produce images in which the internal tissue structure (e.g., the tumor) appears more uniform and exhibits softer contrast than conventional images [8]. In our study, the experienced rater could detect these changes in line with objective quantitative parameters. However, the inexperienced rater was less aware of these subtle changes and was unable to differentiate them in image impression. Therefore, quantitative image quality metrics should complement these subjective impressions and provide valuable context when subjective impressions diverge.

A key contribution of this work is the explicit quantification of edge sharpness using edge steepness, edge width, and edge contrast. These parameters are often observed qualitatively but rarely quantified in DL-accelerated MRI studies. They provide objective support for the perceived improvement of edge sharpness using DLR. Across all sequences and edge detectors, DLR demonstrated significantly higher edge steepness consistent with more abrupt intensity transitions at anatomic boundaries and generally narrower edge widths, providing objective support for the improved sharpness perceived by both raters. Previous studies used edge slope and edge rise distance to quantify image sharpness, as these metrics reflect the signal-intensity transition at the border between hyperintense and hypointense tissues [3], [5]. Seo et al. utilized FWHM to analyze sharpness in DL-accelerated neck MRI [19]. However, previous research has shown that FWHM can underestimate true distances in phantoms of various shapes by up to 28% [49], [50]. Another study noted persistent underestimation of FWHM when spatial resolution exceeded two pixels per diameter [51]. The slight reduction in edge contrast with DLR, combined with the discordance between blur metrics and edge-based measures, underscores that blur perception and edge definition are distinct phenomena. Blur metrics may capture texture smoothing within tissues, whereas edge parameters specifically quantify boundary transitions. This is potentially more clinically relevant for orbital MRI, where interpretation depends on crisp delineation of optic nerve margins, extraocular muscles, and compartment boundaries.

The SSIM and MS-SSIM values were close to 1.0, confirming that the macroscopic structural content was well preserved. In contrast, lower FSIM and WASH values indicated differences in the representation of high-frequency features. This pattern is consistent with DLR strategies that combine data consistency with learned regularization, potentially altering fine texture while maintaining overall fidelity. Although FSIM has been linked to radiologists' image quality ratings, we could not confirm this in our study, which included only two comparison images [31]. Since some similarity measures show little difference between the reconstruction methods, this also implies that specific quantitative measurements, such as those conducted in this study, are necessary.

From a practical standpoint, faster orbital protocols are appealing as orbital imaging is susceptible to motion (blinking, eye movements), and comprehensive evaluation typically requires multiple planes and contrast-enhanced, fat-suppressed acquisitions. By enabling higher acceleration without clear loss of reader confidence in lesion detection or conspicuity, DL-accelerated TSE sequences may help reduce motion-related nondiagnostic examinations and facilitate more robust multi-plane coverage within clinically acceptable scan times. In our study of 25 patients, no nondiagnostic examinations were performed.

This study has several limitations. Firstly, the retrospective design, limited sample size, and a heterogeneous pathology mix, including both benign and malignant pathologies, limit generalizability. However, the consistent quantitative differences between reconstructions suggest similar conclusions would hold in larger cohorts and are independent of the underlying disease. Secondly, CR images served as the reference standard, although no universally accepted ground truth exists for determining which reconstruction technique more faithfully represents anatomy. Thirdly, despite blinding, the characteristic imaging appearance of DLR may have allowed raters to infer sequence type. Fourthly, only two raters were included, as the study's emphasis was on quantitative parameters. Nevertheless, this is in line with other radiological DL studies. Qualitative evaluations are inherently susceptible to individual biases. Fithly, manual SNR/CNR measurements were omitted because these have been extensively reported for Deep Resolve Boost in prior studies. MATLAB-derived SNR and PSNR confirmed superior DLR performance. Lastly, the study was limited to a single manufacturer's DLR technique on a single scanner.

In conclusion, this study provides a comprehensive assessment of accelerated DL-based reconstructions applied to orbital MRI. DLR maintains or improves lesion conspicuity, overall image quality, and edge sharpness, while substantially reducing scan time. However, reduced delineation of internal anatomical structures, i.e., blurriness, warrants caution in interpreting the images.

## Ethical approval

The study was conducted in accordance with the Declaration of Helsinki and its subsequent amendments and approved by the Institutional Ethics Committee of Tübingen (protocol code 549/2024BO2, 3 December 2024).

## CRediT authorship contribution statement

**Christer Ruff:** Writing – review & editing, Writing – original draft, Visualization, Investigation, Formal analysis, Conceptualization. **Till-Karsten Hauser:** Writing – review & editing, Writing – original draft, Visualization, Validation, Project administration, Methodology, Investigation, Formal analysis, Data curation, Conceptualization. **Carina Kelbsch:** Writing – review & editing, Validation. **Frank Paulsen:** Writing – review & editing, Validation. **Deborah Staber:** Writing – review & editing, Visualization, Investigation. **Georg Gohla:** Writing – review & editing, Validation. **Ulrike Ernemann:** Writing – review & editing, Supervision, Resources, Project administration. **Daniel Vogl:** Writing – review & editing, Validation. **Constantin Roder:** Writing – review & editing, Validation.

## Declaration of Competing Interest

The authors declare that they have no known competing financial interests or personal relationships that could have appeared to influence the work reported in this paper.

## Footnotes

Appendix A Supplementary data associated with this article can be found in the online version at doi:10.1016/j.ejro.2026.100761.
