# Supplementary material for: Quantification of edge-enhancing effects using accelerated deep learning reconstructed orbital MRI sequences
Source: Eur J Radiol Open. 2026 May 15;16:100761. doi: 10.1016/j.ejro.2026.100761 (PMC13200132; doi:10.1016/j.ejro.2026.100761)
Supplement: Supplementary file 1 — Supplementary material [file mmc1.docx]

**Supplementary Materials**

**Supplementary Table 1** Qualitative image assessment criteria (5-point Likert scale).

| **Parameter** | **1** | **2** | **3** | **4** | **5** |
| --- | --- | --- | --- | --- | --- |
| Overall image quality | Non-diagnostic. | Poor; marked blurring / noise / artifacts; interpretation hindered but some anatomy visible. | Fair; noticeable blurring / noise / artifacts; interpretation moderately affected. | Good; minimal blurring/noise/artifacts; effective interpretation. | Excellent; exceptionally clear; no relevant blurring / noise / artifacts; optimal for interpretation. |
| Edge sharpness | Not detectable. | Hardly detectable. | Undefined margins. | Well-defined margins. | Excellent sharpness. |
| Tissue blurriness  (higher score = less blur) | Severe; fine detail essentially absent. | Marked; level of detail greatly reduced. | Moderate; some loss of fine detail. | Mostly sharp; fine detail mostly preserved. | Very sharp; fine anatomic detail clearly visible. |
| Overall sharpness | Non-diagnostic; severe blurring in margins of all anatomic structures. | Limited; blurred margins extend beyond diagnostic regions, including stationary structures. | Diagnostic; slight blurring within diagnostic regions, particularly in motion-sensitive structures. | Good; slight blurring outside diagnostic regions. | Excellent; fine sharpness across all anatomic structures. |
| Diagnostic confidence in lesion detection* | Not detected or diagnosed. | Lesion hardly detected; doubtful diagnostic confidence. | Lesion detected / diagnosed with weak confidence. | Lesion detected / diagnosed with moderate confidence. | Lesion confidently detected and diagnosed. |
| Lesion conspicuity  (delineation of primary lesion)* | Entire lesion difficult to delineate. | Contours largely indistinct. | Some margins unclear due to blurring. | Slight blurring in some contours; otherwise clear. | Lesion clearly visualized without blurring. |

All parameters were rated on a 5-point Likert scale (1 = worst, 5 = best). *Lesion-specific criteria were assessed only when a lesion was present.

**Supplementay Table 2** Interrater agreement using Cohen’s Kappa and Kendall’s Tau.

|  | Cohen’s Kappa | Kendall’s Tau b | Kendall’s Tau c |
| --- | --- | --- | --- |
| Overall image quality | | |  |
| T2_CR_ ax | 0.388 | 0.621 | 0.422 |
| T2_DLR_ ax | 0.636 | 0.780 | 0.499 |
| T2_CR_ cor | 0.541 | 0.663 | 0.528 |
| T2_DLR_ cor | 0.712 | 0.815 | 0.666 |
| T1CEfs_CR_ ax | 0.647 | 0.733 | 0.586 |
| T1CEfs_DLR_ ax | 0.773 | 0.880 | 0.614 |
| T1CEfs_CR_ cor | 0.800 | 0.864 | 0.763 |
| T1CEfs_DLR_ cor | 0.878 | 0.981 | 0.640 |
| Edge sharpness | | |  |
| T2_CR_ ax | 0.551 | 0.728 | 0.624 |
| T2_DLR_ ax | 0.632 | 0.883 | 0.691 |
| T2_CR_ cor | 0.711 | 0.819 | 0.672 |
| T2_DLR_ cor | 0.841 | 0.852 | 0.845 |
| T1CEfs_CR_ ax | 0.647 | 0.733 | 0.586 |
| T1CEfs_DLR_ ax | 0.836 | 0.899 | 0.870 |
| T1CEfs_CR_ cor | 0.851 | 0.930 | 0.986 |
| T1CEfs_DLR_ cor | 0.784 | 0.847 | 0.701 |
| Delineation of internal tissue structures (“bluriness”) |  |  |  |
| T2_CR_ ax | 0.649 | 0.849 | 0.830 |
| T2_DLR_ ax | 0.293 | 0.753 | 0.542 |
| T2_CR_ cor | 0.079 | 0.570 | 0.403 |
| T2_DLR_ cor | 0.198 | 0.770 | 0.610 |
| T1CEfs_CR_ ax | 0.100 | 0.656 | 0.538 |
| T1CEfs_DLR_ ax | -0.080 | 0.630 | 0.659 |
| T1CEfs_CR_ cor | 0.072 | 0.687 | 0.605 |
| T1CEfs_DLR_ cor | 0.118 | 0.572 | 0.485 |
| Overall sharpness |  |  |  |
| T2_CR_ ax | 0.694 | 0.894 | 0.850 |
| T2_DLR_ ax | 0.619 | 0.790 | 0.605 |
| T2_CR_ cor | 0.845 | 0.898 | 0.922 |
| T2_DLR_ cor | 0.667 | 0.737 | 0.691 |
| T1CEfs_CR_ ax | 0.841 | 0.852 | 0.845 |
| T1CEfs_DLR_ ax | 0.709 | 0.756 | 0.595 |
| T1CEfs_CR_ cor | 0.770 | 0.830 | 0.858 |
| T1CEfs_DLR_ cor | 0.802 | 0.846 | 0.749 |

ax, axial; cor, coronal; CR, conventional reconstruction; DLR, deep learning reconstruction; CE, contrast-enhanced; fs, fat-saturated.

**Supplementary Table 3** Results and comparison of edge sharpness analysis with conventionally reconstructed (CR) and deep-learning reconstructed (DLR) MRI sequences using different edge detection methods.

|  | Sequence | edge detection method | value  (M ± SD) | Within-Participant Coefficient of Variation, CV [%] | Intraclass Correlation Coefficient (ICC) - consistency | | Intraclass Correlation Coefficient (ICC) - absolute agreement | |
| --- | --- | --- | --- | --- | --- | --- | --- | --- |
|  |  |  |  |  | Single  (CI 95 %) | Average  (CI 95 %) | Single  (CI 95 %) | Average  (CI 95 %) |
| Mean edge steepness | T2_CR_ ax | Roberts | 0.1854 ± 0.0563 | 4.7 | 0.946  (0.898 – 0.974) | 0.946  (0.964 - 0.991) | 0.057  (0.001 – 0.206) | 0.153  (-0.002 – 0.438) |
|  |  | Sobel | 0.1725 ± 0.0576 | 4.9 |  |  |  |  |
|  |  | Canny | 0.1187 ± 0.0618 | 8.1 |  |  |  |  |
|  | T2_DLR_ ax | Roberts | 0.2139 ± 0.0714 | 4.0 | 0.889  (0.799 – 0.945) | 0.960  (0.922 – 0.981) | 0.036  (0 – 0.138) | 0.101  (-0.001 – 0.325) |
|  |  | Sobel | 0.1987 ± 0.0722 | 4.0 |  |  |  |  |
|  |  | Canny | 0.1367 ± 0.0760 | 6.3 |  |  |  |  |
|  | T2_CR_ cor | Roberts | 0.1523 ± 0.0424 | 6.5 | 0.947  (0.899 – 0.974) | 0.982  (0.964 – 0.991) | 0.079  (0.001 - 0,270) | 0.205  (0.003 – 0.526) |
|  |  | Sobel | 0.1426 ± 0.0448 | 6.7 |  |  |  |  |
|  |  | Canny | 0.0950 ± 0.0516 | 8.8 |  |  |  |  |
|  | T2_DLR_ cor | Roberts | 0.1746 ± 0.0700 | 9.4 | 0.808  (0.668 – 0.903) | 0.927  (0.858 – 0.965) | 0.113  (-0.004 – 0.349) | 0.276  (-0.011 – 0.616) |
|  |  | Sobel | 0.1626 ± 0.0711 | 9.0 |  |  |  |  |
|  |  | Canny | 0.1114 ± 0.0682 | 7.2 |  |  |  |  |
|  | T1CEfs_CR_ ax | Roberts | 0.1497 ± 0.0692 | 3.7 | 0.849  (0.732 – 0.925) | 0.944  (0.891 – 0.974) | 0.023  (0 - 0.091) | 0.065  (-0.001 – 0.231) |
|  |  | Sobel | 0.1411 ± 0.0450 | 3.8 |  |  |  |  |
|  |  | Canny | 0.0957 ± 0.0514 | 3.3 |  |  |  |  |
|  | T1CEfs_DLR_ ax | Roberts | 0.1802 ± 0.0682 | 5.2 | 0.807  (0.666 – 0.902) | 0.926  (0.857 – 0.965) | 0.035  (-0.001 - 0.135) | 0.098  (-0.004 – 0.318) |
|  |  | Sobel | 0.1685 ± 0.0692 | 5.0 |  |  |  |  |
|  |  | Canny | 0.1129 ± 0.0710 | 3.7 |  |  |  |  |
|  | T1CEfs_CR_ cor | Roberts | 0.1379 ± 0.0493 | 4.2 | 0.827  (0.696 – 0.913) | 0.935  (0.873 – 0.969) | 0.019  (-0.001 – 0.078) | 0.056  (-0.002 – 0.203) |
|  |  | Sobel | 0.1279 ± 0.0511 | 4.1 |  |  |  |  |
|  |  | Canny | 0.0762 ± 0.0510 | 5.5 |  |  |  |  |
|  | T1CEfs_DLR_ cor | Roberts | 0.1782 ± 0.0809 | 5.4 | 0.778  (0.621 – 0.886) | 0.913  (0.831 – 0.959) | 0.025  (-0.001 - -0.001) | 0.071  (-0.004 – 0.248) |
|  |  | Sobel | 0.1642 ± 0.0824 | 5.1 |  |  |  |  |
|  |  | Canny | 0.0951 ± 0.0749 | 6.2 |  |  |  |  |
| Mean edge width | T2_CR_ ax | Roberts | 3.8138 ± 0.0708 | 1.9 | 0.779  (0.622 – 0.886) | 0.913  (0.832 – 0.959) | 0.126  (-0.006 - 0.376) | 0.301  (-0.017 - 0,644) |
|  |  | Sobel | 3.6449 ± 0.0577 | 1.6 |  |  |  |  |
|  |  | Canny | 3.4974 ± 0.0780 | 2.2 |  |  |  |  |
|  | T2_DLR_ ax | Roberts | 3.7639 ± 0.2013 | 5.3 | 0.813  (0.675 – 0.906) | 0.929  (0.862 – 0.966) | 0.365  (0.001 - 0.692) | 0.633  (0.002 – 0.871) |
|  |  | Sobel | 3.6041 ± 0.1542 | 4.3 |  |  |  |  |
|  |  | Canny | 3.4111 ± 0.1068 | 3.1 |  |  |  |  |
|  | T2_CR_ cor | Roberts | 3.7236 ± 0.0651 | 1.7 | 0.781  (0.627 – 0.888) | 0.915  (0.834 – 0.960) | 0.121  (-0.005 - 0.366) | 0.291  (-0.016 – 0.633) |
|  |  | Sobel | 3.5500 ± 0.0498 | 1.4 |  |  |  |  |
|  |  | Canny | 3.4366 ± 0.0685 | 2.0 |  |  |  |  |
|  | T2_DLR_ cor | Roberts | 3.4846 ± 0.3266 | 9.4 | 0.843  (0.723 – 0.922) | 0.942  (0.887 – 0.972) | 0.753  (0.438 - 0.893) | 0.901  (0.700 – 0.962) |
|  |  | Sobel | 3.3571 ± 0.3019 | 9.0 |  |  |  |  |
|  |  | Canny | 3.2958 ± 0.1496 | 4.5 |  |  |  |  |
|  | T1CEfs_CR_ ax | Roberts | 3.6730 ± 0.0416 | 1.1 | 0.585  (0.361 – 0.770) | 0.809  (0.629 – 0.910) | 0.043  (-0.006 - 0.160) | 0.118  (-0.018 – 0.363) |
|  |  | Sobel | 3.5175 ± 0.0346 | 1.0 |  |  |  |  |
|  |  | Canny | 3.3992 ± 0.0385 | 1.1 |  |  |  |  |
|  | T1CEfs_DLR_ ax | Roberts | 3.5065 ± 0.1068 | 3.0 | 0.667  (0.464 – 0.821) | 0.857  (0.722 – 0.932) | 0.155  (-0.013 - 0.427) | 0.355  (-0.040 – 0.691) |
|  |  | Sobel | 3.3659 ± 0.1002 | 3.0 |  |  |  |  |
|  |  | Canny | 3.1730 ± 0.0596 | 1.9 |  |  |  |  |
|  | T1CEfs_CR_ cor | Roberts | 3.6342 ± 0.0489 | 1.3 | 0.485  (0.244 – 0.703) | 0.739  (0.492 – 0.876) | 0.041  (-0.008 - 0.152) | 0.113  (-0.026 -0.349) |
|  |  | Sobel | 3.4666 ± 0.0402 | 1.2 |  |  |  |  |
|  |  | Canny | 3.3409 ± 0.0438 | 1.3 |  |  |  |  |
|  | T1CEfs_DLR_ cor | Roberts | 3.3802 ± 0.1138 | 3.4 | 0.486  (0.245 – 0.703) | 0.739  (0.494 – 0.877) | 0.239  (-0.008 - 0.521) | 0.486  (-0.023 – 0.765) |
|  |  | Sobel | 3.2468 ± 0.1311 | 4.0 |  |  |  |  |
|  |  | Canny | 3.1620 ± 0.0662 | 2.1 |  |  |  |  |
| Mean edge contrast | T2_CR_ ax | Roberts | 0.5494 ± 0.0192 | 3.5 | 0.859  (0.748 – 0.930) | 0.948  (0.899 – 0.975) | 0.035  (-0.001 - 0.134) | 0.098  (-0.002 – 0.318) |
|  |  | Sobel | 0.5186 ± 0.0192 | 3.7 |  |  |  |  |
|  |  | Canny | 0.3549 ± 0.0254 | 7.2 |  |  |  |  |
|  | T2_DLR_ ax | Roberts | 0.5362 ± 0.0186 | 3.5 | 0.717  (0.533 – 0.851) | 0.884  (0.774 – 0.945) | 0.026  (-0.002 - 0.102) | 0.073  (-0.006 – 0.254) |
|  |  | Sobel | 0.5096 ± 0.0151 | 3.0 |  |  |  |  |
|  |  | Canny | 0.3523 ± 0.0230 | 6.5 |  |  |  |  |
|  | T2_CR_ cor | Roberts | 0.5235 ± 0.0246 | 4.7 | 0.912  (0.837 – 0.957) | 0.969  (0.939 – 0.985) | 0.044  (0 - 0.167) | 0.122  (0- 0.375) |
|  |  | Sobel | 0.4956 ± 0.0237 | 4.8 |  |  |  |  |
|  |  | Canny | 0.3269 ± 0.0240 | 7.3 |  |  |  |  |
|  | T2_DLR_ cor | Roberts | 0.4697 ± 0.0422 | 9.0 | 0.758  (0.591 – 0.875) | 0.904  (0.813 – 0.954) | 0.109  (-0.006 – 0.339) | 0.268  (-0.018 – 0.606) |
|  |  | Sobel | 0.4471 ± 0.0385 | 8.6 |  |  |  |  |
|  |  | Canny | 0.3113 ± 0.0204 | 6.6 |  |  |  |  |
|  | T1CEfs_CR_ ax | Roberts | 0.4745 ± 0.0138 | 2.9 | 0.860  (0.750 – 0.930) | 0.949  (0.900 – 0.976) | 0.014  (0 – 0.058) | 0.041  (-0.001 – 0.156) |
|  |  | Sobel | 0.4480 ± 0.0132 | 3.0 |  |  |  |  |
|  |  | Canny | 0.2983 ± 0.0088 | 2.9 |  |  |  |  |
|  | T1CEfs_DLR_ ax | Roberts | 0.4330 ± 0.0210 | 4.9 | 0.849  (0.731 – 0.924) | 0.944  (0.891 – 0.973) | 0.036  (-0.001 – 0.138) | 0.101  (-0.002 – 0.325) |
|  |  | Sobel | 0.4137 ± 0.0192 | 4.7 |  |  |  |  |
|  |  | Canny | 0.2748 ± 0.0129 | 4.7 |  |  |  |  |
|  | T1CEfs_CR_ cor | Roberts | 0.4648 ± 0.0126 | 2.7 | 0.694  (0.501 – 0.838) | 0.872  (0.751 – 0.939) | 0.008  (-0.001 – 0.032) | 0.022  (-0.002 – 0.090) |
|  |  | Sobel | 0.4339 ± 0.0122 | 2.8 |  |  |  |  |
|  |  | Canny | 0.2543 ± 0.0109 | 4.3 |  |  |  |  |
|  | T1CEfs_DLR_ cor | Roberts | 0.4522 ± 0.0205 | 4.5 | 0.750  (0.580 – 0.870) | 0.900  (0.805 – 0.953) | 0.019  (-0.001 – 0.078) | 0.056  (-0.004 – 0.203) |
|  |  | Sobel | 0.4259 ± 0.0175 | 4.1 |  |  |  |  |
|  |  | Canny | 0.2429 ± 0.0175 | 7.2 |  |  |  |  |

Data are presented as mean ± standard deviation. M, mean; SD, standard deviation; CV, Coefficient of Variation; ICC, Intraclass Correlation Coefficient; ax, axial; cor, coronal; CR, conventional reconstruction; DLR, deep learning reconstruction; CE, contrast-enhanced; fs = fat-saturated.
